# Fibromyalgia and Risk of Alzheimer’s DiseaseRelated Dementia: A Nationwide Bidirectional Case–Control Study

**DOI:** 10.3390/geriatrics11030061

**Published:** 2026-05-18

**Authors:** Eli Magen, Israel Magen, Eugene Merzon, Ilan Green, Avivit Golan-Cohen, Shlomo Vinker, Ariel Israel

**Affiliations:** 1 Leumit Health Services, Tel Aviv-Yafo 6473817, Israel; emarzon@leumit.co.il (E.M.); igreen@leumit.co.il (I.G.); agolanchoen@leumit.co.il (A.G.-C.); svinker@leumit.co.il (S.V.); aisrael@leumit.co.il (A.I.); 2 Department of Medicine A, Assuta Ashdod University Medical Center, Ben-Gurion University of the Negev, Ashdod 7747629, Israel; 3 Faculty of Health Sciences, Ben-Gurion University of the Negev, Beer Sheva 8410501, Israel; isr.magen@gmail.com; 4 Urology Department, Assuta Ashdod University Medical Center, Ben-Gurion University of the Negev, Ashdod 7747629, Israel; 5 Adelson School of Medicine, Ariel University, Ariel 4070000, Israel; 6 Department of Family Medicine, Gray Faculty of Medical and Health Sciences, Tel Aviv University, Tel Aviv-Yafo 6997801, Israel; 7 Department of Epidemiology and Preventive Medicine, School of Public Health, Faculty of Medical and Health Sciences, Tel Aviv University, Tel Aviv-Yafo 6139001, Israel

**Keywords:** fibromyalgia, Alzheimer disease, dementia, neurodegenerative diseases, comorbidity, case–control studies, epidemiology, risk factors, electronic health records, longitudinal studies

## Abstract

**Background/Objectives:** To evaluate the association between fibromyalgia and dementia, with emphasis on temporal directionality and Alzheimer’s disease-related dementia. **Methods:** We conducted a nationwide, population-based matched case–control study including 9232 patients with fibromyalgia and 46,160 age- and sex-matched controls. Diagnoses in the Leumit Health Services database are recorded using a hybrid coding scheme that combines ICD-9-CM and WHO ICD-10 codes; the specific codes used to ascertain fibromyalgia and each dementia subtype are listed in the Methods. Outcomes were assessed within two predefined windows: up to 20 years before and up to 10 years after fibromyalgia diagnosis. Alzheimer disease-related dementia was defined as the primary outcome. Multivariable logistic regression was used to estimate odds ratios (ORs) with 95% confidence intervals (CIs). **Results:** During the 20 years preceding fibromyalgia diagnosis, no increased dementia prevalence was observed; Alzheimer disease-related dementia was less frequent among fibromyalgia patients (0.16% vs. 0.31%; absolute difference −0.15 percentage points; OR 0.52, 95% CI 0.31–0.89). In contrast, during the 10 years following diagnosis, fibromyalgia was associated with a higher prevalence of Alzheimer’s disease-related dementia (1.43% vs. 0.99%; absolute difference +0.44 percentage points; OR 1.45, 95% CI 1.18–1.78). No consistent associations were found for other dementia subtypes, which should be interpreted as exploratory given low event counts. **Conclusions:** Fibromyalgia is associated with a higher prevalence of Alzheimer’s disease-related dementia in the years following diagnosis, with no evidence of pre-diagnostic elevation. Although this temporal pattern argues against reverse causation, the prevalence-based design and residual confounding preclude causal inference. Fibromyalgia should be regarded as a potential risk marker for subsequent Alzheimer-related neurodegeneration rather than a demonstrated causal factor.

## 1. Introduction

Fibromyalgia is a chronic pain disorder characterized by widespread musculoskeletal pain, fatigue, sleep disturbance, and cognitive complaints [1]. Although traditionally classified as a rheumatologic condition, increasing evidence suggests that fibromyalgia involves central nervous system dysregulation, including altered pain processing, neuroinflammation, and functional brain changes [2,3]. Cognitive symptoms—often termed “fibro fog”—commonly include impairments in attention, memory, and executive function; however, their long-term clinical significance remains uncertain [4,5].

Dementia, particularly Alzheimer’s disease, represents a growing global health burden [6,7]. Established risk factors include cardiometabolic disease, depression, chronic inflammation, and sleep disturbance—features frequently observed in fibromyalgia populations [8,9,10]. These shared characteristics have led to the hypothesis that fibromyalgia may be associated with increased risk of subsequent neurodegenerative disease [11,12].

Recent epidemiological evidence supports this possibility. A nationwide population-based cohort study demonstrated that fibromyalgia is associated with a significantly increased risk of incident dementia, including Alzheimer’s, vascular, and nonvascular subtypes [13]. Proposed mechanisms include systemic and central inflammation, central sensitization, and structural brain changes such as reduced hippocampal volume [13]. However, key uncertainties remain. Specifically, it is unclear whether fibromyalgia precedes and contributes to dementia risk, represents an early manifestation of neurodegeneration, or reflects shared confounding factors.

The temporal relationship between fibromyalgia and dementia is therefore critical. Some studies suggest that cognitive dysfunction in fibromyalgia is distinct from neurodegenerative processes, whereas others propose overlapping or progressive mechanisms [14,15]. Distinguishing between these hypotheses requires study designs capable of addressing reverse causation and diagnostic bias.

In this study, we conducted a large nationwide population-based analysis to evaluate the association between fibromyalgia and dementia, with a focus on Alzheimer’s disease-related dementia. Using a bidirectional temporal design, we assessed dementia prevalence both before and after fibromyalgia diagnosis. We hypothesized that fibromyalgia would be associated with increased dementia burden emerging after diagnosis, but not before. Our findings are interpreted as identifying fibromyalgia as a potential risk marker for subsequent Alzheimer-related dementia, rather than as evidence of a causal relationship.

## 2. Materials and Methods

### 2.1. Study Design and Data Source

We conducted a nationwide, population-based matched case–control study using electronic medical records from Leumit Health Services (LHS), a large health maintenance organization in Israel, with longitudinal follow-up. The LHS database contains comprehensive patient-level information, including demographic characteristics, laboratory measurements, outpatient and inpatient diagnoses, medication records, and hospitalization data. Diagnoses in the LHS database are recorded using a hybrid coding scheme. The majority of diagnoses are coded using ICD-9-CM, reflecting the system’s historical configuration. Dementia and several other neuropsychiatric conditions are coded using the World Health Organization ICD-10 classification (which uses the F00–F03 series for dementia, in contrast to ICD-10-CM, which reclassifies Alzheimer’s disease under G30). Both code sets coexist in the database. To avoid ambiguity, every code used in the present analysis is listed explicitly in Section 2.3 and Section 2.5 below.

Access to the dataset is restricted due to institutional and privacy regulations. However, de-identified data may be made available upon reasonable request, subject to approval by the data custodians.

### 2.2. Diagnostic Coding Framework

Because the present analysis depends critically on which International Classification of Diseases (ICD) version is used for which condition, we summarise the LHS coding framework here in a single dedicated subsection. The LHS database employs a hybrid scheme. The majority of clinical diagnoses—including all somatic comorbidities tabulated in Table 1—are recorded using ICD-9-CM, reflecting the historical configuration of the LHS diagnostic dictionary. A defined subset of neuropsychiatric conditions, including dementia and Alzheimer disease, is captured using the World Health Organization ICD-10 classification (the F00–F03 series for dementia, distinct from the ICD-10-CM scheme used in the United States, which reclassifies Alzheimer disease under G30). Both code sets coexist in the same database and can be queried using standard structured queries.

This hybrid configuration is dictionary-driven rather than user-selectable: the clinician records a diagnosis from the LHS-supplied pick list, and the recorded code follows whichever classification the dictionary assigns to that diagnosis. For dementia, the dictionary issues ICD-10 codes (F00.x for Alzheimer disease, F01 for vascular dementia) alongside legacy ICD-9-CM codes (331.0, 290.x, 294.1x for Alzheimer-related dementias, 290.4x for vascular dementia, 331.1 for frontotemporal dementia, 331.82 and 331.820 for Lewy-body/Parkinsonism dementias). For fibromyalgia, the dictionary historically issues ICD-9-CM 729.1 with the LHS-specific subcode 729.10. The implications of this asymmetry for fibromyalgia ascertainment are discussed in Section 2.3 below. The complete list of codes used to ascertain each outcome appears in Section 2.5.

### 2.3. Study Population

Patients with fibromyalgia were identified using ICD-9-CM code 729.1 (“Myalgia and myositis, unspecified”, the LHS legacy code historically used for fibromyalgia in this database) and the LHS subcode 729.10 (“Fibromyalgia”). Both qualifying encounters were required to be with a board-certified rheumatologist, occurring on different calendar days, with the second encounter at least 30 days after the first. The index date was defined as the first recorded diagnosis of fibromyalgia.

To our knowledge, this LHS case-finding algorithm has not been previously validated in the LHS database itself or in another Israeli population-based dataset. We therefore acknowledge that residual misclassification of fibromyalgia status remains possible despite the dual-rheumatologist requirement, and we discuss this as a limitation in Section 4.5. The two-encounter, rheumatologist-only criterion is intended as a pragmatic mitigation: it excludes one-off coding artifacts and screening codes used by primary-care clinicians without specialist confirmation, which together account for a substantial proportion of false-positive fibromyalgia entries in administrative data.

A natural further question, given the hybrid coding framework described in Section 2.2, is whether ICD-10 M79.7 (the ICD-10 designation for fibromyalgia) was considered for case ascertainment. We retained ICD-9-CM 729.1 + 729.10 rather than migrating to M79.7 for two reasons. First, in contrast to the dementia subset, where the LHS diagnostic dictionary issues ICD-10 codes natively, fibromyalgia in the LHS dictionary is captured under the legacy ICD-9-CM 729.x family with the local subcode 729.10. M79.7 is not consistently populated for cases predating the partial ICD-10 migration of selected diagnostic categories, which would create artefactual discontinuity at the migration boundary if used as the case-finding code. Second, switching mid-cohort would have introduced a calendar-time gradient in case ascertainment that is impossible to disentangle from the temporal exposure-outcome window of interest. We therefore acknowledge explicitly that the present study uses asymmetric coding systems for exposure (ICD-9-CM) and outcome (predominantly ICD-10), and we treat this asymmetry as a structural feature of the LHS data rather than a free analytic choice. Robustness of the findings to alternative case definitions remains a target for replication in databases without this asymmetry.

Each fibromyalgia case was matched with up to five control individuals without fibromyalgia based on age, sex, and calendar year. Controls were required to be free of fibromyalgia at the index date.

The final study cohort included 9232 patients with fibromyalgia and 46,160 matched controls.

### 2.4. Baseline Variables

Baseline characteristics were assessed at or prior to the index date and included demographic variables (age, sex, and ethnic group [Arab, general Jewish, and ultra-orthodox Jewish]), socioeconomic status, body mass index (BMI), and smoking status.

Laboratory parameters included serum creatinine, glucose levels, hemoglobin A1c, and hemoglobin.

Comorbid conditions were identified using ICD-9-CM and WHO ICD-10 codes (as appropriate to the diagnostic category) and comprised cardiovascular diseases (atrial fibrillation, hypertension, ischemic heart disease), metabolic disorders (diabetes mellitus, dyslipidemia), autoimmune and endocrine conditions (connective tissue diseases, Graves’ disease, Hashimoto’s thyroiditis), respiratory diseases (asthma, chronic obstructive pulmonary disease), liver disease (cirrhosis), renal disease (chronic renal disease), malignancies (solid tumors and hematological malignancies), and alcoholism.

### 2.5. Outcome Definitions and Temporal Framework

Dementia diagnoses were identified from the LHS diagnostic dictionary using the following codes: Alzheimer disease-related dementia (primary outcome)—WHO ICD-10 codes F00, F00.0, F00.00, F00.01, F00.03, F00.04, F00.1, F00.11, and F00.13, together with ICD-9-CM codes 331.0 (Alzheimer’s disease), 290.0 (senile dementia, uncomplicated), 290.10 (presenile dementia, uncomplicated), 290.12 (presenile dementia with delusional features), 290.13 (presenile dementia with depressive features), 290.20 and 290.21 (senile dementia with delusional/depressive features), 290.3 (senile dementia with delirium), 290.8, 290.9, and 294.10–294.11 (dementia in conditions classified elsewhere); vascular dementia—WHO ICD-10 F01, F01.1, F01.91, F01.93, F01.94, with ICD-9-CM 290.4, 290.42, and 290.43; frontotemporal dementia—ICD-9-CM 331.1 and 331.19; dementia with Lewy bodies/dementia in Parkinsonism—ICD-9-CM 331.82 and 331.820; other and unspecified dementia (sensitivity analyses only)—WHO ICD-10 F02, F02.8, and F03. Alzheimer disease-related dementia (all subtypes combined) was defined as the primary outcome, while other dementia subtypes were considered secondary outcomes and analyzed as exploratory. Throughout the manuscript, the primary outcome bundle described above—which combines specific Alzheimer codes (F00.x and ICD-9-CM 331.0) with the broader 290.x and 294.1x family that captures uncertain or mixed dementias—is referred to as *“Alzheimer disease and related or unspecified dementias (broad definition).”* To address concerns that this broad bundle may inflate the outcome and weaken subtype specificity, we additionally pre-specified a sensitivity outcome restricted to ICD-10 F00.x and ICD-9-CM 331.0 only (excluding 290.x and 294.1x). This narrow sensitivity outcome is reported in Section 3.4 alongside the other prespecified sensitivity analyses. For readability, the conventional shorter label “Alzheimer disease–related dementia” is retained in the title, abstract, results tables, and discussion of this manuscript; this usage refers to the broad primary outcome defined above. The narrow-outcome sensitivity analysis in Section 3.4 confirms that the broad and narrow definitions yield equivalent conclusions, so this labeling choice does not affect the substantive findings. Readers should nonetheless interpret the “Alzheimer disease–related dementia” outcome as inclusive of nonspecific senile, presenile, and dementia-in-conditions-classified-elsewhere codes rather than as confirmed subtype-specific Alzheimer disease.

To assess temporal relationships, dementia prevalence was evaluated within two predefined periods: (1) a pre-diagnostic period of up to 20 years prior to the index date, and (2) a post-diagnostic period of up to 10 years following the index date. The main analysis focused on dementia occurring after fibromyalgia diagnosis, with additional analyses examining pre-diagnostic prevalence.

### 2.6. Statistical Analysis

Baseline characteristics were compared using appropriate parametric or nonparametric tests, depending on the data distribution.

Associations between fibromyalgia and dementia were estimated using odds ratios (ORs) with 95% confidence intervals (CIs) derived from logistic regression models. Multivariable models adjusted for predefined covariates, including demographic characteristics, socioeconomic status, and clinically relevant comorbidities. Age, sex, and calendar year of the index date were retained as covariates in the multivariable models in addition to being used for matching, in line with current recommendations for matched case–control analyses. Depression was not included as a covariate in the primary multivariable model. This decision was made a priori on causal-inference grounds: depression is a well-established downstream consequence of fibromyalgia and an independent risk factor for Alzheimer-related dementia, placing it on the plausible causal pathway between the two conditions. Adjustment for a mediator partially blocks the indirect effect of the exposure operating through that mediator, biasing the total-effect estimate toward the null [16]. The role of depression is therefore examined empirically through a prespecified subgroup analysis stratified by depression status (Section 3.4) rather than through covariate adjustment.

To evaluate temporal relationships and reduce reverse causation, a bidirectional design was applied, examining dementia prevalence both before (up to 20 years) and after (up to 10 years) the index date. The primary analysis focused on post-diagnostic dementia.

To address multiple comparisons in subtype analyses, *p*-values were adjusted using the Benjamini–Hochberg false discovery rate (FDR). Odds ratio estimates and confidence intervals are not reported for cells in which either group contained fewer than five events; these are flagged as “insufficient data” and treated as exploratory.

Prespecified sensitivity analyses included (1) lag-time analyses excluding dementia diagnoses within 1–2 years after the index date, (2) alternative models with expanded comorbidity adjustment, (3) adjustment for healthcare utilization to mitigate surveillance bias, and (4) restriction analyses excluding patients with major neurological or psychiatric conditions. Healthcare utilization was operationalized as the number of primary-care outpatient encounters and all-cause hospitalizations recorded in the year preceding the index date, both modeled as continuous variables.

Subgroup analyses were conducted by age, sex, depression status, and cardiometabolic comorbidity burden, with interaction terms used to assess effect modification.

Missing data were handled using complete-case analysis when <5% missingness, and multiple imputation otherwise. Model assumptions were evaluated using standard diagnostic procedures, including assessment of multicollinearity, goodness-of-fit, and influential observations.

Potential biases were addressed through matching on age, sex, and calendar year (selection bias), multivariable adjustment (confounding), a bidirectional temporal design and adjustment for healthcare utilization (surveillance bias), and lag-time and pre-diagnostic analyses (reverse causation).

All tests were two-sided, with statistical significance defined as *p* < 0.05 after appropriate adjustment.

A formal a priori sample size calculation was not performed because the sample size is fixed by the available LHS cohort; we therefore report a post hoc power statement for the primary outcome. With 9232 cases and 46,160 controls, the observed prevalences in the post-diagnostic window were 1.43% (cases) versus 0.99% (controls), corresponding to an absolute difference of +0.44 percentage points. Using the standard two-proportion z-test at α = 0.05 (two-sided), this design has approximately 94% power to detect the observed difference. The minimum detectable absolute difference between groups under the same parameters, with 80% power, is approximately 0.33 percentage points, well below the observed +0.44 percentage points. Power for non-Alzheimer dementia subtypes was substantially lower due to the small absolute event counts, consistent with the “insufficient data” designation applied to those rows in Table 2 and Table 3, and is the primary reason we restrict formal interpretation to the Alzheimer-related outcome.

### 2.7. Ethical Approval

The study protocol was approved by the institutional review board (IRB) of the participating organization. Due to the retrospective nature of the study and the use of de-identified data, the requirement for informed consent was waived.

### 2.8. Use of Generative Artificial Intelligence

Generative artificial intelligence tools were used solely for language editing and improvement of manuscript clarity. No AI tools were used for data generation, analysis, or interpretation.

### 2.9. STROBE Reporting Compliance

This study was designed and reported in accordance with the *Strengthening the Reporting of Observational Studies in Epidemiology (STROBE)* guidelines for case–control studies. All recommended items—including study design, setting, participants, variables, bias mitigation, study size justification, statistical methods, and limitations—have been addressed. A completed STROBE checklist is provided as Supplementary Material.

## 3. Results

### 3.1. Baseline Characteristics

The study population included 9232 individuals with fibromyalgia and 46,160 matched controls. By design, the groups were comparable with respect to age and sex (Table 1).

Compared with controls, patients with fibromyalgia exhibited a higher burden of clinical risk factors, including:

- Increased body mass index;
- Higher prevalence of smoking;
- Greater frequency of cardiometabolic conditions (e.g., diabetes, hypertension, dyslipidemia);
- Higher prevalence of autoimmune, respiratory, and oncologic comorbidities.

These differences indicate a substantially higher baseline comorbidity burden among individuals with fibromyalgia and should be borne in mind throughout the interpretation of the results, as residual confounding by unmeasured aspects of this broader risk profile (including medication exposure, sleep disturbance, habitual physical activity, and depression severity) cannot be excluded.

### 3.2. Dementia Prevalence Prior to Fibromyalgia Diagnosis

During the 20-year period preceding fibromyalgia diagnosis, no increased prevalence of dementia was observed among individuals who later developed fibromyalgia compared with controls (Table 2). The full subcode breakdown is provided in Supplementary Table S1.

When Alzheimer disease-related dementia was analyzed as an aggregated outcome, fibromyalgia patients had a significantly lower prevalence than controls (0.16% vs. 0.31%; absolute difference −0.15 percentage points; OR 0.52, 95% CI 0.31–0.89). As discussed in Section 4.2, this inverse pre-diagnostic association should not be interpreted as evidence of a protective effect; alternative explanations such as diagnostic competition with prodromal cognitive impairment, differential healthcare engagement, and survivorship are explored there.

No significant associations were observed for vascular dementia, frontotemporal dementia, dementia with Lewy bodies, or Parkinsonism-associated dementia, and event counts in these subtypes were too small to support reliable estimation.

These findings indicate the absence of a detectable pre-diagnostic dementia signal in individuals who subsequently develop fibromyalgia.

### 3.3. Dementia Prevalence Following Fibromyalgia Diagnosis

During the 10-year period following fibromyalgia diagnosis, a higher prevalence of Alzheimer disease-related dementia was observed among fibromyalgia patients compared with controls (1.43% vs. 0.99%; absolute difference +0.44 percentage points; OR 1.45, 95% CI 1.18–1.78) (Table 3). The full subcode breakdown is provided in Supplementary Table S2.

Within the broad Alzheimer disease-related outcome, point estimates were elevated for both the early-onset (F00.0) and late-onset (F00.1) subcategories, shown in Table 3. By contrast, event counts for vascular dementia, frontotemporal dementia, dementia with Lewy bodies, and Parkinsonism-associated dementia were too small to support reliable estimation and are flagged as “insufficient data” in the full ICD-code breakdown (Supplementary Table S2).

These results suggest that the observed association is most readily detectable for Alzheimer disease-related dementia rather than generalized across dementia subtypes, although the apparent specificity should be interpreted cautiously: low event counts for non-Alzheimer subtypes limit statistical power, and administrative coding of dementia subtypes is itself prone to misclassification.

### 3.4. Sensitivity, Subgroup, and Robustness Analyses

The association between fibromyalgia and Alzheimer’s disease-related dementia remained consistent across all prespecified sensitivity analyses.

In lag-time analyses, exclusion of dementia cases occurring within the first year after fibromyalgia diagnosis yielded similar results (OR 1.42, 95% CI 1.15–1.76), as did exclusion within the first two years (OR 1.39, 95% CI 1.12–1.73), indicating that reverse causation is unlikely to explain the observed association.

Additional adjustment for cardiometabolic, psychiatric, and inflammatory comorbidities did not significantly alter the estimates (OR 1.41, 95% CI 1.14–1.75). Similarly, adjustment for healthcare utilization measures, including outpatient visits and hospitalizations, yielded consistent results (OR 1.43, 95% CI 1.16–1.77). Restriction analyses excluding patients with major neurological or psychiatric disorders also demonstrated comparable results (OR 1.38, 95% CI 1.10–1.72).

Subgroup analyses showed consistent associations across all examined strata. The association was similar in individuals aged <65 years and ≥65 years (OR 1.38, 95% CI 1.05–1.81 vs. OR 1.47, 95% CI 1.19–1.82; *p* = 0.41), in males and females (OR 1.42, 95% CI 1.05–1.93 vs. OR 1.46, 95% CI 1.17–1.82; *p* = 0.67), in individuals with and without depression (OR 1.51, 95% CI 1.18–1.94 vs. OR 1.42, 95% CI 1.12–1.79; *p* = 0.29), and in those with high versus low cardiometabolic burden (OR 1.48, 95% CI 1.19–1.85 vs. OR 1.39, 95% CI 1.10–1.75; *p* = 0.35).

Analyses using multiple imputation for missing data produced results comparable to complete-case analyses (OR 1.44 vs. 1.46). Model diagnostics confirmed adequate model performance, with no evidence of multicollinearity (variance inflation factors < 2.5), acceptable goodness-of-fit (Hosmer–Lemeshow *p* = 0.21), and no influential observations.

The pre-specified narrow sensitivity outcome (Section 2.5), restricted to ICD-10 F00.x and ICD-9-CM 331.0 only, yielded conclusions consistent with the primary analysis. In the post-diagnostic window, narrow-outcome events occurred in 99 of 9232 cases (1.07%) and in 342 of 46,160 controls (0.74%), yielding an absolute difference of +0.33 percentage points and an OR of 1.43 (95% CI, 1.14–1.79). In the pre-diagnostic window, narrow-outcome events occurred in 11 of 9232 cases (0.12%) and 105 of 46,160 controls (0.23%), for an absolute difference of −0.11 percentage points and an OR of 0.51 (95% CI 0.27–0.95). Both the direction and the magnitude of the bidirectional association are preserved when the outcome is restricted to specific Alzheimer codes, indicating that the inclusion of 290.x and 294.1x non-specific codes is not the driver of the observed pattern. A complete summary of all sensitivity, subgroup, and robustness analyses is provided in Supplementary Table S3.

### 3.5. Competing Mortality

Because fibromyalgia patients in our cohort entered with a higher baseline burden of cardiometabolic and oncologic comorbidities (Table 1), differential mortality during follow-up could, in principle, bias the post-diagnostic dementia comparison in either direction. We therefore enumerated all-cause mortality during the 10-year post-diagnostic window for both groups. Among the 9232 cases, 356 (3.86%) died within the 10-year window, compared with 2137 of 46,160 controls (4.63%); the absolute difference was −0.77 percentage points, and the crude odds ratio for death among cases versus controls was 0.83 (95% CI 0.74–0.93; χ^2^ test, *p* = 0.001). Contrary to the expectation generated by the baseline comorbidity profile, all-cause mortality during follow-up was therefore modestly but significantly lower among cases than among matched controls.

Reasoning explicitly through the direction of the resulting bias: because cases survived longer than controls, they had, on average, more cumulative follow-up time during which a dementia diagnosis could be recorded. This pattern can *inflate*, rather than attenuate, the observed case–control difference in dementia prevalence. The observed post-diagnostic OR of 1.45 (95% CI 1.18–1.78) should therefore be regarded as a plausibly upper-bound rather than a conservative estimate of the underlying association, and the true magnitude of the post-diagnostic association is plausibly somewhat smaller than 1.45. We note, however, that this inflation acts on the magnitude of the post-diagnostic effect and does not generate a spurious positive association where none would otherwise exist; in particular, it does not explain the bidirectional pattern observed in this study (no pre-diagnostic elevation and, if anything, a lower pre-diagnostic prevalence in cases), which continues to argue against reverse causation as the dominant explanation for the post-diagnostic signal. Several mechanisms may contribute to the unexpectedly lower mortality among cases: fibromyalgia patients engage with primary care more often than the general population, which favours earlier detection and treatment of competing chronic disease; the cohort is predominantly female (86.8%) and middle-aged at index (mean 47.6 years), a stratum with comparatively low baseline mortality risk; and the two-rheumatologist case definition implicitly conditions on survival to the second qualifying encounter, introducing an immortal-time-like selection in favour of healthier cases. The direction and approximate magnitude of the residual inflationary bias on the dementia OR cannot be quantified without person-time data and formal competing-risk modeling, and we therefore retain the limitation in Section 4.5.

## 4. Discussion

In this large nationwide population-based study, fibromyalgia was associated with an increased prevalence of Alzheimer disease-related dementia emerging after diagnosis, whereas no increased dementia burden—and in fact a lower prevalence—was observed during the two decades preceding fibromyalgia diagnosis. This bidirectional temporal pattern provides evidence against reverse causation and is most consistent with fibromyalgia acting as a risk marker for, rather than an early manifestation of, subsequent Alzheimer-related neurodegeneration. The prevalence-based design and the residual confounding inherent in observational data preclude any stronger causal claim.

### 4.1. Interpretation in the Context of Previous Literature

Our findings extend and refine prior epidemiological evidence. A large nationwide cohort study demonstrated that fibromyalgia is associated with a substantially increased risk of incident dementia (adjusted hazard ratio 2.77), including Alzheimer’s, vascular, and nonvascular subtypes [13]. While that study established an association, it did not address temporal directionality. By incorporating a bidirectional design, our study provides critical insight, demonstrating that the association emerges specifically after fibromyalgia diagnosis.

Importantly, we observed an apparent specificity for Alzheimer’s disease-related dementia, in contrast to prior research suggesting increased risk across multiple dementia subtypes [13,17,18]. This difference may reflect methodological distinctions, including our aggregation of Alzheimer-related diagnoses to reduce misclassification and our focus on temporal prevalence rather than incidence. However, the apparent subtype specificity should be interpreted cautiously. Administrative coding of dementia subtypes is known to be imperfect, with non-trivial misclassification between Alzheimer-related, vascular, and mixed dementias and between early- and late-onset categories. The relative reliability of coding for Alzheimer-related dementia—the most common and most familiar diagnostic category in routine practice—may itself contribute to the appearance of specificity, independently of any truly differential biological effect.

### 4.2. Implications for Causality, Bias, and Alternative Explanations

The absence of increased dementia prevalence prior to fibromyalgia diagnosis argues against reverse causation and reduces the likelihood that prodromal dementia is being misclassified as fibromyalgia [19]. It does not, however, exclude the alternative possibility that prodromal cognitive impairment makes accurate ascertainment of the diffuse pain history required for a fibromyalgia diagnosis difficult—a form of diagnostic competition rather than a true protective effect. Differential healthcare engagement may also contribute: patients destined to develop fibromyalgia may engage with primary care more than the general population, but the specific subgroup with early cognitive decline may engage less, and survivorship bias prior to the index date may further depress observed pre-diagnostic prevalence. The inverse direction of the pre-diagnostic estimate (OR 0.52) should therefore not be interpreted as evidence that fibromyalgia is protective; rather, it is most plausibly explained by the absence of true reverse causation combined with these ascertainment effects. Specifically, accurate ascertainment of fibromyalgia requires the clinician to elicit a detailed history of widespread pain, fatigue, sleep disturbance, and tender-point distribution—all of which depend on the patient’s ability to articulate symptom patterns coherently over time. Prodromal cognitive impairment (memory difficulty, reduced verbal fluency, executive dysfunction) directly impedes this history-taking process, making a positive fibromyalgia diagnosis substantially less likely in patients who are already on a dementia trajectory. This is a more specific instance of diagnostic competition than the general healthcare-engagement explanation, and it provides a parsimonious mechanism for the apparently “protective” pre-diagnostic OR of 0.52 without invoking biological protection.

Similarly, the inverse pre-diagnostic association makes it unlikely that increased healthcare utilization alone explains the post-diagnostic findings; if surveillance bias were dominant, elevated dementia prevalence would be expected both before and after diagnosis [20,21]. Even so, residual confounding by factors that we could not measure or could only measure imperfectly—including medication exposure (in particular long-term opioid, gabapentinoid, and benzodiazepine use), sleep disturbance, habitual physical activity, depression severity, and cognitive reserve—may contribute to the observed association. In this context, fibromyalgia in our cohort is best understood as a marker of a broader risk profile rather than as an isolated exposure.

A specific point concerns the role of depression. Depression is a well-established downstream consequence of fibromyalgia and an independent risk factor for Alzheimer-related dementia, placing it on the plausible causal pathway between the two conditions rather than acting as a simple confounder. We therefore did not adjust for depression in the primary multivariable model, because adjustment for a mediator would partially block the indirect effect of fibromyalgia operating through depression and bias the total-effect estimate toward the null [16]. The empirical question of whether the observed association is driven by depression is addressed instead by the prespecified subgroup analysis stratified by depression status (Section 3.4), which showed essentially identical effect sizes in patients with and without depression (OR 1.51 vs. OR 1.42; *p* for interaction = 0.29). Depression is therefore neither a sufficient confounder nor a necessary intermediary for the observed association, although it likely contributes as one of several pathways linking fibromyalgia to subsequent Alzheimer-related dementia.

Together, these findings support fibromyalgia as a potential risk marker for subsequent Alzheimer-related neurodegeneration, rather than a consequence of early cognitive decline. They do not establish a causal relationship, and the modest absolute difference in prevalence (0.44 percentage points) should be borne in mind when considering clinical implications.

### 4.3. Biological Mechanisms

The pathways summarised below are speculative; they are presented to motivate future mechanistic research and cannot be tested directly with the present administrative data. Although causal mechanisms cannot be established in this observational study, several biologically plausible pathways may underlie the observed association. Fibromyalgia has been linked to systemic and central nervous system inflammation, including elevated proinflammatory cytokines, which are increasingly implicated in Alzheimer’s pathogenesis [22,23,24]. Chronic pain and central sensitization may reflect altered neural network function and increased neural “noise,” potentially predisposing to cognitive vulnerability [25,26]. More broadly, chronic pain is associated with brain structural changes overlapping with Alzheimer’s disease [27].

Neuroimaging studies have demonstrated reduced hippocampal volume in fibromyalgia patients, a key region affected early in Alzheimer’s disease [28,29]. In addition, common comorbidities in fibromyalgia—including sleep disturbance, depression, and cardiometabolic disease—are independently associated with increased dementia risk [13,30,31].

Whether fibromyalgia itself contributes directly to neurodegeneration or reflects cumulative exposure to these risk factors remains an important question for future research.

### 4.4. Clinical and Research Implications

Given the modest effect size and the observational design of this study, the clinical implications are correspondingly modest. Patients with fibromyalgia appear to constitute a population at modestly increased risk for Alzheimer’s disease-related dementia, which clinicians may wish to take into account in routine clinical care. We do not, however, advocate fibromyalgia-specific cognitive screening protocols based on these data; cognitive evaluation in fibromyalgia patients should follow general guidelines for older adults until confirmatory longitudinal evidence is available. Second, the present findings highlight fibromyalgia as a potential model for studying interactions between chronic pain, neuroinflammation, and neurodegeneration [23,32,33].

Future studies should focus on longitudinal incidence analyses, integration of neuroimaging and biomarker data, and mechanistic investigations to clarify causality and identify modifiable pathways.

### 4.5. Limitations

This study has several limitations. Diagnoses of fibromyalgia and dementia were based on administrative coding, which may introduce misclassification. Our fibromyalgia case definition required the LHS fibromyalgia subcode 729.10 and two separate encounters with board-certified rheumatologists, but the parent ICD-9-CM code 729.1 is not fibromyalgia-specific and, to our knowledge, no published validation study of this exact LHS algorithm is available; exposure misclassification, therefore, cannot be excluded, and the dual-rheumatologist requirement is a pragmatic mitigation rather than a substitute for formal validation. ICD-10 M79.7 was not used because fibromyalgia is captured in the LHS legacy rheumatology dictionary through 729.1/729.10 during the study period (see Section 2.3); this introduces an asymmetric exposure/outcome coding configuration that is a structural feature of the data rather than a free analytic choice. Dementia ascertainment—and, in particular, the assignment of dementia subtype—is also vulnerable to misclassification, with non-trivial coding errors between Alzheimer-related, vascular, and mixed dementias, and between early- and late-onset categories, well documented in routine electronic health record data. Because the primary outcome aggregates specific Alzheimer codes with nonspecific senile, presenile, and dementia-in-conditions-classified-elsewhere codes, the apparent signal should be interpreted as Alzheimer disease-related or unspecified dementia rather than as confirmed subtype-specific Alzheimer disease. Detailed clinical data, including neuropsychological testing and biomarker information, were not available. The observational design precludes causal inference, and residual confounding cannot be ruled out despite multivariable adjustment; in particular, medication use (especially long-term opioid, gabapentinoid, and benzodiazepine exposure), sleep disturbance, habitual physical activity, and depression severity were not measured with sufficient granularity to fully account for their effects. Due to limitations in precise onset timing, prevalence-based analyses were used; however, consistent results across temporal windows and sensitivity analyses support the robustness of the findings.

Because dementia outcomes were assessed as cumulative prevalence within predefined temporal windows rather than incident events, results may be influenced by survival bias and healthcare utilization patterns, and the prevalence-based design does not allow estimation of incidence rates. However, the bidirectional temporal design mitigates concerns regarding reverse causation. In addition, the competing risk of mortality may have affected the observed associations, and as enumerated in Section 3.5, all-cause mortality during the 10-year post-diagnostic window was modestly but significantly lower among cases than controls (3.86% vs. 4.63%; OR 0.83, 95% CI 0.74–0.93; *p* = 0.001), a pattern that may inflate rather than attenuate the observed case–control difference in dementia prevalence; the observed post-diagnostic OR of 1.45 should therefore be regarded as a plausibly upper-bound estimate of the underlying association, although the bidirectional temporal pattern (absence of any pre-diagnostic elevation) continues to argue against reverse causation as the dominant explanation. Without person-time data and formal competing-risk modeling, the residual magnitude of this bias cannot be quantified. Residual confounding, particularly by cognitive reserve and medication use, cannot be excluded.

## 5. Conclusions

Fibromyalgia was associated with a higher prevalence of Alzheimer’s disease-related dementia in the 10 years following fibromyalgia diagnosis, with no evidence of pre-diagnostic elevation. The bidirectional temporal pattern argues against reverse causation, but the prevalence-based design and residual confounding preclude causal inference. These findings support the interpretation of fibromyalgia as a potential risk marker—rather than a demonstrated causal contributor—for subsequent Alzheimer-related neurodegeneration, and warrant further longitudinal studies to clarify incidence, trajectories, and potential mediating factors.

## Figures and Tables

**Table 1:** Demographic and clinical characteristics of study subjects at baseline.

	Case *n* = 9232	Control *n* = 46,160	*p*
**Female *n* (%)**	8017 (86.8%)	40,085 (86.8%)	1
**Age (years)**	47.6 ± 12.4	47.6 ± 12.4	0.967
**BMI (kg/m^2^)**	28.5 ± 6.1	27.6 ± 5.8	<0.001
**Smoker *n* (%)**	1979 (21.44%)	7841 (16.99%)	<0.001
**Ethnic group—Arab *n* (%)**	2327 (25.2%)	8982 (19.5%)	<0.001
**Ethnic group—General *n* (%)**	5693 (61.7%)	30,065 (65.1%)	<0.001
**Ethnic group—Jewish Ultra-orthodox *n* (%)**	1212 (13.1%)	7113 (15.4%)	<0.001
**Socio-economic status (1–20)**	8.53 ± 3.50	8.58 ± 3.52	0.216
**Creatinine (mg/dL)**	0.70 ± 0.15	0.72 ± 0.26	<0.001
**Glucose (mg/dL)**	97.8 ± 26.9	98.3 ± 28.0	0.119
**Hemoglobin A1c (%)**	5.72 ± 0.95	5.79 ± 1.09	<0.001
**Hemoglobin (g/dL)**	13.2 ± 1.4	13.1 ± 1.4	<0.001
**Atrial fibrillation *n* (%)**	90 (0.97%)	445 (0.96%)	0.907
**Alcoholism *n* (%)**	21 (0.23%)	93 (0.20%)	0.615
**Asthma *n* (%)**	855 (9.26%)	2211 (4.79%)	<0.001
**Cirrhosis**	7 (0.076%)	50 (0.108%)	0.477
**Connective tissue disease *n* (%)**	872 (9.45%)	872 (1.89%)	<0.001
**COPD *n* (%)**	467 (5.06%)	1176 (2.55%)	<0.001
**Diabetes *n* (%)**	1028 (11.14%)	4109 (8.90%)	<0.001
**Dyslipidemia *n* (%)**	2937 (31.8%)	12,045 (26.1%)	<0.001
**Graves’ disease *n* (%)**	214 (2.32%)	791 (1.71%)	0.001
**Hashimoto’s disease *n* (%)**	752 (8.15%)	2797 (6.06%)	<0.001
**Hematological malignancy *n* (%)**	51 (0.55%)	209 (0.45%)	0.211
**Hypertension *n* (%)**	2075 (22.5%)	9303 (20.2%)	<0.001
**Ischemic heart disease *n* (%)**	282 (3.05%)	1026 (2.22%)	<0.001
**Chronic renal disease *n* (%)**	54 (0.58%)	319 (0.69%)	0.295
**Solid tumor *n* (%)**	374 (4.05%)	1382 (2.99%)	<0.001

**Table 2:** Prevalence of dementia in the 20 years before fibromyalgia diagnosis (aggregated outcomes).

Outcome	Case *n* = 9232	Control *n* = 46,160	Absolute Diff. (pp)	*p*	FDR q	OR [95% CI]
**Alzheimer disease-related dementia (all subtypes; primary outcome)**	15 (0.16%)	143 (0.31%)	−0.15	0.014	0.084	0.52 [0.31–0.89]
**Early-onset Alzheimer-related dementia (F00.0)**	6 (0.07%)	24 (0.05%)	+0.02	0.623	1.000	Insufficient data
**Late-onset Alzheimer-related dementia (F00.1)**	2 (0.02%)	32 (0.07%)	−0.05	0.107	0.414	Insufficient data

Footnote: Insufficient data = either group contained fewer than five events; subtype-specific analyses are exploratory and statistically unreliable. Absolute differences are expressed in percentage points (pp). The full subcode breakdown is provided in Supplementary Table S1.

**Table 3:** Prevalence of dementia in the 10 years after fibromyalgia diagnosis (aggregated outcomes).

Outcome	Case *n* = 9232	Control *n* = 46,160	Absolute Diff. (pp)	*p*	FDR q	OR [95% CI]
**Alzheimer disease-related dementia (all subtypes; primary outcome)**	132 (1.43 %)	456 (0.99 %)	+0.44	<0.001	0.004	1.45 [1.18–1.78]
**Early-onset Alzheimer-related dementia (F00.0)**	42 (0.46 %)	133 (0.29 %)	+0.17	0.014	0.083	1.58 [1.09–2.25]
**Late-onset Alzheimer-related dementia (F00.1)**	54 (0.59 %)	182 (0.39 %)	+0.20	0.014	0.081	1.49 [1.08–2.03]

Footnote: Insufficient data = either group contained fewer than five events; subtype-specific analyses are exploratory and statistically unreliable. Absolute differences are expressed in percentage points (pp). The full subcode breakdown is provided in Supplementary Table S2.

## Data Availability

The data supporting the findings of this study are available from Leumit Health Services, but restrictions apply. Data are available from the corresponding author upon reasonable request and with permission from Leumit Health Services.

## Supplementary Materials

The following supporting information can be downloaded at: https://www.mdpi.com/article/10.3390/geriatrics11030061/s1, Table S1. Full ICD-code breakdown of dementia diagnoses recorded in the 20 years before fibromyalgia diagnosis; Supplementary Table S2. Full ICD-code breakdown of dementia diagnoses recorded in the 10 years after fibromyalgia diagnosis; Table S3. Sensitivity, subgroup, and robustness analyses for the primary outcome (post-diagnostic Alzheimer disease–related dementia); Table S4: STROBE Statement—Checklist of items that should be included in reports of case–control studies.

## Abbreviations

AD—Alzheimer’s disease; BMI—Body mass index; CI—Confidence interval; COPD—Chronic obstructive pulmonary disease; FDR—False discovery rate; ICD-9-CM—International Classification of Diseases, Ninth Revision, Clinical Modification; ICD-10—International Classification of Diseases, Tenth Revision (WHO version); IRB—Institutional Review Board; LHS—Leumit Health Services; OR—Odds ratio.
