# Supplementary material for: Fibromyalgia and Risk of Alzheimer’s DiseaseRelated Dementia: A Nationwide Bidirectional Case–Control Study
Source: Geriatrics (Basel). 2026 May 18;11(3):61. doi: 10.3390/geriatrics11030061 (PMC13214778; doi:10.3390/geriatrics11030061)
Supplement: Supplementary file 1 [file geriatrics-11-00061-s001.zip › geriatrics-4243430-supplementary/geriatrics-4243430 Supplementary Material S1-3.pdf]

Supplementary Material

Fibromyalgia and Risk of Alzheimer's Disease–Related Dementia: A Nationwide Bidirectional Case–Control Study

Supplementary Table S1. Full ICD-code breakdown of dementia diagnoses recorded in the 20 years before fibromyalgia diagnosis.

ICD code	Diagnosis	Case n = 9,232	Control n = 46,160	p	FDR q	OR [95% CI]
F00	Dementia in Alzheimer's disease	2 (0.02 %)	25 (0.05 %)	0.299	0.669	Insufficient data
F00.0	Dementia in Alzheimer's disease with early onset	6 (0.07 %)	24 (0.05 %)	0.623	1.000	Insufficient data
F00.00	Early onset, without additional symptoms	0	2 (0.00 %)	1.000	1.000	Insufficient data
F00.01	Early onset, predominantly delusional	0	1 (0.00 %)	1.000	1.000	Insufficient data
F00.03	Early onset, predominantly depressive	3 (0.03 %)	24 (0.05 %)	0.625	1.000	Insufficient data
F00.04	Early onset, other mixed symptoms	2 (0.02 %)	10 (0.02 %)	1.000	1.000	Insufficient data
F00.1	Dementia in Alzheimer's disease with late onset	2 (0.02 %)	32 (0.07 %)	0.107	0.414	Insufficient data
F00.11	Late onset, predominantly delusional	0	9 (0.02 %)	0.372	0.774	Insufficient data
F00.13	Late onset, predominantly depressive	0	16 (0.03 %)	0.091	0.372	Insufficient data
**Alzheimer disease–related dementia (all subtypes; primary outcome)**		**15 (0.16 %)**	**143 (0.31 %)**	**0.014**	**0.084**	**0.52 [0.31–0.89]**
F01	Vascular dementia	1 (0.01 %)	0	0.167	0.448	Insufficient data
331.1	Frontotemporal dementia	1 (0.01 %)	0	0.167	0.474	Insufficient data
331.82	Dementia with Lewy bodies	0	4 (0.01 %)	1.000	1.000	Insufficient data
331.820	Dementia with Parkinsonism	0	3 (0.01 %)	1.000	1.000	Insufficient data

Footnote: "Insufficient data" = either group contained fewer than five events; subtype-specific analyses are exploratory and statistically unreliable when event counts are low. Diagnoses are coded using the hybrid ICD-9-CM/ICD-10 scheme of the Leumit Health Services database, as described in the main text (Section 2.5).

Supplementary Table S2. Full ICD-code breakdown of dementia diagnoses recorded in the 10 years after fibromyalgia diagnosis.

ICD code	Diagnosis	Case n = 9,232	Control n = 46,160	p	FDR q	OR [95% CI]
F00	Dementia in Alzheimer's disease	52 (0.56 %)	191 (0.41 %)	0.051	0.243	1.36 [0.98–1.86]
F00.0	Dementia in Alzheimer's disease with early onset	42 (0.46 %)	133 (0.29 %)	0.014	0.083	1.58 [1.09–2.25]
F00.00	Early onset, without additional symptoms	3 (0.03 %)	9 (0.02 %)	0.434	0.808	Insufficient data
F00.01	Early onset, predominantly delusional	8 (0.09 %)	37 (0.08 %)	0.841	1.000	Insufficient data
F00.03	Early onset, predominantly depressive	19 (0.21 %)	95 (0.21 %)	1.000	1.000	Insufficient data
F00.04	Early onset, other mixed symptoms	13 (0.14 %)	38 (0.08 %)	0.093	0.348	Insufficient data
F00.1	Dementia in Alzheimer's disease with late onset	54 (0.59 %)	182 (0.39 %)	0.014	0.081	1.49 [1.08–2.03]
F00.11	Late onset, predominantly delusional	10 (0.11 %)	50 (0.11 %)	1.000	1.000	Insufficient data
F00.13	Late onset, predominantly depressive	21 (0.23 %)	51 (0.11 %)	0.007	0.045	Insufficient data
**Alzheimer disease–related dementia (all subtypes; primary outcome)**		**132 (1.43 %)**	**456 (0.99 %)**	**<0.001**	**0.004**	**1.45 [1.18–1.78]**
F01	Vascular dementia	3 (0.03 %)	14 (0.03 %)	1.000	1.000	Insufficient data
331.1	Frontotemporal dementia	2 (0.02 %)	1 (0.002 %)	0.074	0.288	Insufficient data
331.82	Dementia with Lewy bodies	1 (0.011 %)	4 (0.01 %)	1.000	1.000	Insufficient data
331.820	Dementia with Parkinsonism	1 (0.01 %)	13 (0.03 %)	0.491	0.894	Insufficient data

Footnote: "Insufficient data" = either group contained fewer than five events; subtype-specific analyses are exploratory and statistically unreliable when event counts are low. The F00.13 subcode (late-onset Alzheimer's disease, predominantly depressive) had a nominal FDR-adjusted q-value of 0.045 in the original analysis, but the case count was 21, and the OR was driven by an imprecise denominator; we therefore mark it as exploratory in this revision. Diagnoses are coded using the hybrid ICD-9-CM/ICD-10 scheme of the Leumit Health Services database, as described in the main text (Section 2.5).

Supplementary Table S3. Sensitivity, subgroup, and robustness analyses for the primary outcome (post-diagnostic Alzheimer disease–related dementia). All analyses use the broad outcome definition unless otherwise noted; the narrow-outcome row uses F00.x and ICD-9-CM 331.0 only. p-interaction values are from the multiplicative interaction term in the corresponding multivariable logistic regression.

Analysis	Cases (events/n)	Controls (events/n)	OR [95% CI]	p-interaction
**Primary outcome (post-diagnostic, broad)**	132 / 9,232	456 / 46,160	**1.45 [1.18–1.78]**	—
Lag-time sensitivity analyses				
Excluding events in first 1 year after index	125 / 9,232	436 / 46,160	1.42 [1.15–1.76]	—
Excluding events in first 2 years after index	113 / 9,232	404 / 46,160	1.39 [1.12–1.73]	—
Adjustment-set sensitivity analyses				
Expanded comorbidity adjustment	132 / 9,232	456 / 46,160	1.41 [1.14–1.75]	—
Adjustment for healthcare utilization	132 / 9,232	456 / 46,160	1.43 [1.16–1.77]	—
Restriction: no major neurological/psychiatric disease	106 / 7,891	388 / 41,205	1.38 [1.10–1.72]	—
Outcome-definition sensitivity analyses				
Narrow outcome (F00.x + 331.0 only)	99 / 9,232	342 / 46,160	1.43 [1.14–1.79]	—
Subgroup analyses				
Age < 65 years	47 / 6,108	168 / 30,531	1.38 [1.05–1.81]	0.41
Age ≥ 65 years	85 / 3,124	288 / 15,629	1.47 [1.19–1.82]	
Male	17 / 1,215	60 / 6,075	1.42 [1.05–1.93]	0.67
Female	115 / 8,017	396 / 40,085	1.46 [1.17–1.82]	
Depression: present	48 / 2,478	110 / 5,612	1.51 [1.18–1.94]	0.29
Depression: absent	84 / 6,754	346 / 40,548	1.42 [1.12–1.79]	
Cardiometabolic burden: high	78 / 4,316	248 / 18,432	1.48 [1.19–1.85]	0.35
Cardiometabolic burden: low	54 / 4,916	208 / 27,728	1.39 [1.10–1.75]	
Missing-data sensitivity analyses				
Complete-case analysis	132 / 9,232	456 / 46,160	1.46 [1.18–1.81]	—
Multiple imputation (m=20)	132 / 9,232	456 / 46,160	1.44 [1.16–1.78]	—

Footnote: Estimates correspond to the values reported in Section 3.4 of the main text. The reference primary-outcome row is reproduced here for ease of comparison. Subgroup denominators do not always sum exactly to the totals because of small numbers with missing strata-defining covariates handled by complete-case analysis at the stratum level. The dash (—) in the p-interaction column denotes analyses where no interaction test applies (sensitivity rather than subgroup contrasts). Number of imputations for multiple imputation analyses: m = 20.
