# Supplementary material for: Fibromyalgia and Risk of Alzheimer’s DiseaseRelated Dementia: A Nationwide Bidirectional Case–Control Study
Source: Geriatrics (Basel). 2026 May 18;11(3):61. doi: 10.3390/geriatrics11030061 (PMC13214778; doi:10.3390/geriatrics11030061)
Supplement: Supplementary file 1 [file geriatrics-11-00061-s001.zip › geriatrics-4243430-supplementary/geriatrics-4243430-supplementary S4.pdf]

Table S4. STROBE Statement — Checklist of items that should be included in reports of case–control studies

Manuscript ID: geriatrics-4243430

Title: "Fibromyalgia and Risk of Alzheimer Disease–Related Dementia: A Nationwide Bidirectional Case–Control Study"

Section / Topic	Item No.	Recommendation	Reported on page/section
Title and abstract	1	(a) Indicate the study's design with a commonly used term in the title or the abstract	Title ("matched case–control study"); Abstract
		(b) Provide in the abstract an informative and balanced summary of what was done and what was found	Abstract (Background/Objectives, Methods, Results, Conclusions)
**Introduction**			
Background/rationale	2	Explain the scientific background and rationale for the investigation being reported	Section 1, paragraphs 1–3
Objectives	3	State-specific objectives, including any prespecified hypotheses	Section 1, final paragraph
**Methods**			
Study design	4	Present key elements of study design early in the paper	Section 2.1; Abstract (Methods)
Setting	5	Describe the setting, locations, and relevant dates, including periods of recruitment, exposure, follow-up, and data collection	Sections 2.1, 2.3; temporal windows defined in Section 2.5
Participants	6	(a) Give the eligibility criteria, and the sources and methods of case ascertainment and control selection. Give the rationale for the choice of cases and controls	Section 2.3 (Study Population), including exposure definition, two-rheumatologist criterion, and explicit acknowledgment that the algorithm has not been externally validated
		(b) For matched studies, give matching criteria and the number of controls per case	Section 2.3 (matching on age, sex, and calendar year; up to 5 controls per case)
Variables	7	Clearly define all outcomes, exposures, predictors, potential confounders, and effect modifiers. Give diagnostic criteria, if applicable	Exposure: Section 2.3; Baseline covariates: Section 2.4; Outcomes (broad and narrow definitions): Section 2.5; ICD coding framework: Section 2.2

Data sources/measurement	8*	For each variable of interest, give sources of data and details of methods of assessment (measurement). Describe comparability of assessment methods if there is more than one group	Sections 2.1, 2.3, 2.4, 2.5; hybrid ICD-9-CM / WHO ICD-10 coding framework summarised in Section 2.2
Bias	9	Describe any efforts to address potential sources of bias	Section 2.6 (matching, multivariable adjustment, bidirectional design, healthcare-utilization adjustment, lag-time analyses); Section 3.5 (empirical competing-mortality direction-of-bias analysis); Section 4.5 (Limitations)
Study size	10	Explain how the study size was arrived at	Section 2.6 (final paragraph): sample size fixed by the available LHS cohort; post-hoc power statement and minimum detectable effect calculation provided
Quantitative variables	11	Explain how quantitative variables were handled in the analyses. If applicable, describe which groupings were chosen and why	Section 2.6 (continuous covariates modeled in logistic regression; healthcare utilization modeled as continuous); Section 3.4 (age dichotomized at 65 years; high vs low cardiometabolic burden for subgroup analyses)
Statistical methods	12	(a) Describe all statistical methods, including those used to control for confounding	Section 2.6
		(b) Describe any methods used to examine subgroups and interactions	Section 2.6 (subgroup analyses with multiplicative interaction terms); Section 3.4; Supplementary Table S3
		(c) Explain how missing data were addressed	Section 2.6 (complete-case analysis when <5% missingness; multiple imputation otherwise); robustness reported in Section 3.4 and Supplementary Table S3
		(d) If applicable, explain how matching of cases and controls was addressed	Section 2.6 (matching variables retained as covariates in the multivariable models, consistent with current recommendations for matched case–control analyses)
		(e) Describe any sensitivity analyses	Section 2.6 (prespecified analyses listed); Section 3.4 (results); Supplementary Table S3 (lag-time, expanded adjustment, healthcare utilization, restriction, narrow-outcome, subgroup, and missing-data analyses)

**Results**			
Participants	13*	(a) Report numbers of individuals at each stage of study—e.g., numbers potentially eligible, examined for eligibility, confirmed eligible, included in the study, completing follow-up, and analyzed	Section 2.3 (cohort construction); Section 3.1 (9,232 fibromyalgia cases and 46,160 matched controls)
		(b) Give reasons for non-participation at each stage	Not applicable: population-based EHR cohort; all eligible records meeting the case definition (and matched controls) were retained
		(c) Consider use of a flow diagram	A flow diagram was not used; cohort construction is described in narrative form in Section 2.3
Descriptive data	14*	(a) Give characteristics of study participants (e.g., demographic, clinical, social) and information on exposures and potential confounders	Table 1; Section 3.1
		(b) Indicate number of participants with missing data for each variable of interest	Section 2.6 (missingness was below the 5% threshold for the primary covariates, and complete-case analysis was used; variable-level counts not separately tabulated)
Outcome data	15*	Report numbers in each exposure category, or summary measures of exposure	Tables 2 and 3 (aggregated main outcomes); Supplementary Tables S1 and S2 (full ICD-code breakdown for pre- and post-diagnostic windows)
Main results	16	(a) Give unadjusted estimates and, if applicable, confounder-adjusted estimates and their precision (e.g., 95% confidence interval). Make clear which confounders were adjusted for and why they were included	Sections 3.2 and 3.3 (multivariable-adjusted ORs with 95% CIs); Section 2.6 (rationale for the covariate set, including the a priori decision to treat depression as a mediator rather than a confounder)
		(b) Report category boundaries when continuous variables were categorized	Section 3.4 (age dichotomized at 65 years for subgroup analyses; cardiometabolic burden categorized as high vs low for subgroup analyses; specific cut-points are operational and not reported as primary findings)
		(c) If relevant, consider translating estimates of relative risk into absolute risk for a meaningful time period	Sections 3.2 and 3.3 (absolute prevalence differences in percentage points, alongside ORs); Abstract

Other analyses	17	Report other analyses done—e.g., analyses of subgroups and interactions, and sensitivity analyses	Section 3.4 (sensitivity, subgroup, and robustness analyses, including the prespecified narrow-outcome sensitivity analysis); Section 3.5 (competing-mortality analysis); Supplementary Table S3
**Discussion**			
Key results	18	Summarise key results with reference to study objectives	Section 4, opening paragraph; Section 5 (Conclusions)
Limitations	19	Discuss limitations of the study, taking into account sources of potential bias or imprecision. Discuss both direction and magnitude of any potential bias	Section 4.5 (Limitations), including misclassification of exposure and outcome, asymmetric ICD coding, residual confounding, and competing mortality; Section 3.5 reports the empirical direction-of-bias analysis for competing mortality, concluding that the observed lower mortality among cases may inflate rather than attenuate the post-diagnostic association
Interpretation	20	Give a cautious overall interpretation of results, considering objectives, limitations, multiplicity of analyses, results from similar studies, and other relevant evidence	Sections 4.1–4.4 (literature context, causality and alternative explanations, biological mechanisms, clinical implications)
Generalisability	21	Discuss the generalisability (external validity) of the study results	Section 4.4 (clinical implications framed as modest and not warranting fibromyalgia-specific screening); Section 4.5 (LHS-specific case-finding and asymmetric coding noted as limits to direct transportability; replication in databases without this coding asymmetry identified as a priority for future work)
**Other information**			
Funding	22	Give the source of funding and the role of the funders for the present study and, if applicable, for the original study on which the present article is based	Funding statement (back matter): the study received no external funding

*** information separately for cases and controls.**

Note: An Explanation and Elaboration article discusses each checklist item and gives methodological background and published examples of transparent reporting. The STROBE checklist is best used in conjunction with this article (freely available on the Web sites of PLoS Medicine at http://www.plosmedicine.org/, Annals of Internal Medicine at http://www.annals.org/, and Epidemiology at http://www.epidem.com/). Information on the STROBE Initiative is available at www.strobe-statement.org.
